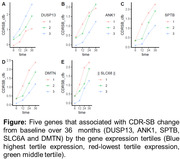# Inflammation and cytoskeleton pathway gene expression relates to rate of clinical progression in individuals with Alzheimers disease

**DOI:** 10.1002/alz.090296

**Published:** 2025-01-09

**Authors:** Gurkan Bebek, Hyun Jo Kim, Penelope Benchek, Lynn M. Bekris, James B Leverenz, William S. Bush, Jagan A. Pillai

**Affiliations:** ^1^ Case Western Reserve University, Cleveland, OH USA; ^2^ Department of Population and Quantitative Health Sciences, Case Western Reserve University, Cleveland, OH USA; ^3^ Cleveland Clinic Lerner Research Institute, Cleveland, OH USA; ^4^ Cleveland Clinic Lou Ruvo Center for Brain Health, Cleveland, OH USA; ^5^ Cleveland Institute for Computational Biology, Department of Population and Quantitative Health Sciences, Case Western Reserve University, Cleveland, OH USA

## Abstract

**Background:**

Inflammatory changes are a key element of Alzheimer’s disease (AD)pathophysiology. However, there is lack of clarity regarding key gene drivers of the cytokines identified as playing a role in clinical AD progression and their relationship to AD biomarkers to understand their clinical role.

**Method:**

We investigated transcriptomic datasets from Alzheimer’s Disease Neuroimaging Initiative (ADNI) and Harvard Brain Tissue Resource Center (HBTRC) to evaluate regulatory gene networks in human AD. We hypothesized that key inflammatory genes are differentially upregulated in AD, predicting clinical progression. ADNI peripheral blood transcriptomic data with cerebrospinal fluid (CSF) AD biomarker confirmed diagnosis was available for 30 AD dementia, 95 MCI, and 41 controls. This was next compared against HBTRC dorsolateral pre‐frontal cortex tissue gene expression data from 376 AD dementia and 173 controls with neuropathology confirmed diagnosis. The association between differentially expressed genes (DEGs) and change in cognition from baseline in ADNI, measured by clinical dementia rating scale ‐sum of boxes (CDR‐SB) at baseline 6, 12, 24 and 36 months using a linear mixed model (LMM) with random intercepts.

**Result:**

We identified 170 DEGs between AD and controls common to ADNI and HBTRC. Among the DEGs, canonical inflammation signaling pathway genes were enriched. Using an FDR cutoff of 0.15, five genes (DUSP13, ANK1, SPTB, SLC6A and DMTN) were associated with significant CDR‐SB change from baseline in MCI and AD. Four of these genes were highly correlated (r2 > 0.8) and were involved in the Spectrin‐associated cytoskeleton pathway (GO:0014731). Among them, increased expression of DUSP13 in AD compared to controls (log2FC = 0.17) was associated with an increased decline in cognition (P = 0.001). An increased expression of ANK1 was related to reduced decline in cognition (P = 0.002) and ANK1 expression was lower in AD compared to MCI (log2FC = ‐0.16). CDR‐SB models incorporating gene expression for these significant genes were equivalent to or better than models incorporating CSF pTau, Aβ42, or MRI hippocampal volume when evaluated by Akaike information criterion.

**Conclusion:**

Key peripheral inflammation and cytoskeletal gene expression relates to clinical progression in human AD. Validation of these results is an important future direction.